# Generation of a new bioluminescent model for visualisation of mammary tumour development in transgenic mice

**DOI:** 10.1186/1471-2407-12-209

**Published:** 2012-05-30

**Authors:** Agnieszka M Zagozdzon, Patrick O’Leary, John J Callanan, John Crown, William M Gallagher, Radoslaw Zagozdzon

**Affiliations:** 1 Cancer Biology and Therapeutics Group, UCD School of Biomolecular and Biomedical Science, UCD Conway Institute, University College Dublin, Belfield, Dublin 4, Ireland; 2 UCD School of Veterinary Medicine & Conway Institute, Belfield, Dublin 4, Ireland; 3 Molecular Therapeutics for Cancer Ireland (MTCI), c/o National Institute for Cellular Biotechnology, Dublin City University, Glasnevin, Dublin 9, Ireland

**Keywords:** Transgenic mice, MMTV promoter, Luciferase, Bioluminescence, Breast cancer, ***in vivo*** imaging

## Abstract

**Background:**

Numerous transgenic models have been generated to study breast cancer. However, despite many advantages, traditional transgenic models for breast cancer are also burdened with difficulties in early detection and longitudinal observation of transgene-induced tumours, which in most cases are randomly located and occur at various time points. Methods such as palpation followed by mechanical measurement of the tumours are of limited value in transgenic models. There is a crucial need for making these previously generated models suitable for modern methods of tumour visualisation and monitoring, e.g. by bioluminescence-based techniques. This approach was successfully used in the current study.

**Results:**

A new mouse strain (MMTV-Luc2 mice) expressing Luc2 luciferase primarily in mammary tissue in females, with low-level background expression in internal organs, was generated and bred to homozygosity. After these mice were intercrossed with MMTV-PyVT mice, all double transgenic females developed mammary tumours by the age of 10 weeks, the localisation and progression of which could be effectively monitored using the luminescence-based ***in vivo*** imaging. Luminescence-based readout allowed for early visualisation of the locally overgrown mammary tissue and for longitudinal evaluation of local progression of the tumours. When sampled ***ex vivo*** at the age of 10 weeks, all tumours derived from MMTV-Luc2PyVT females displayed robust bioluminescent signal.

**Conclusions:**

We have created a novel transgenic strain for visualisation and longitudinal monitoring of mammary tumour development in transgenic mice as an addition and/or a new and more advanced alternative to manual methods. Generation of this mouse strain is vital for making many of the existing mammary tumour transgenic models applicable for ***in vivo*** imaging techniques.

## Background

Transgenic cancer models are invaluable research tools because they recapitulate the entire process from the initial genetic events in normal cells to metastatic disease. Therefore, transgenic mice are ideally suited for studies on the role of oncogenes in carcinogenesis and on the progression of the tumours to invasion and metastasis. Transgenes can be induced to express in the mouse mammary gland under the control of various transgenic promoters, such as MMTV-LTR, WAP, C(3)1 and BLG, which have particular characteristics in expression patterns and other biological aspects. Among these, mouse mammary tumour virus promoter/enhancer (MMTV-LTR) has been most used (reviewed in [1]), since this promoter provides expression of a gene of interest in the mammary epithelium in both non-lactating and lactating females. In the last three decades, numerous transgenic mouse models of breast cancer have been generated by manipulating growth factors and their receptors, as well as cell cycle regulators and other signal transduction mediators. For instance, the MMTV promoter has been used for expression in transgenic animals of various oncogenes, such as Neu/ErbB2/HER2 (reviewed in [2-4]), cyclin D1 [5], Ras [6] or Myc [7]. These molecules have been selected based on the fact that their genes or their products are frequently altered in human breast cancers and studies in these models produced relevant results for breast cancer research. One of the classical transgenic models for breast cancer development involved the mammary gland-specific expression of the polyomavirus middle T antigen (PyVT) expressed under the transcriptional control of the MMTV promoter [8]. In this model, expression of the PyVT oncogene in the breast induced widespread transformation of the mammary epithelium and rapid development of multifocal mammary adenocarcinomas.

Despite the advantages of animal transgenic models for breast cancer, these models are also burdened with certain shortcomings. One of the major obstacles in studies involving transgenic models are problems associated with early detection and monitoring of transgene-induced tumours, which in most cases are randomly located and occur at various time points. Methods such as palpation followed by mechanical measurement of the tumour using callipers are of limited value in transgenic models, although such attempts have been undertaken [9,10]. Theoretically, these difficulties can be overcome by the use of advanced whole-body imaging techniques such as microCT, MRI, PET or SPECT. However, such techniques are laborious, considerably expensive and/or raise significant safety issues. Conversely, non-invasive optical imaging appears to be a feasible and safe alternative to the radiation-based imaging techniques (reviewed in [11-13]). Indeed, introduction of advanced optical imaging techniques for ***in vivo*** imaging of tumour growth and progression in animal models have revolutionised ***in vivo*** cancer studies.

Optical imaging systems have conventionally played a significant role in molecular imaging of gene reporters, molecular targets and receptors in preclinical studies of cancer. The availability of tomographic techniques has further advanced the use of ***in vivo*** optical imaging systems. Optical imaging agents are typically either bioluminescent or fluorescent in their nature. Currently, bioluminescence readout from cells expressing firefly luciferase is regarded the most sensitive system for optical imaging, especially after the recent development of the next-generation enhancement of firefly luciferase, namely Luc2 [14]. Hence, dramatic progress has been made in visualising different kinds of transplanted tumour tissues in both syngeneic and xenotransplantation models. Nevertheless, generation of transgenic tumour models which are ‘compatible’ with bioluminescence-based imaging is hampered by the necessity for dual expression of both the transgene and the luciferase gene in the tissue of interest. For newly generated models, this can be successfully achieved on a case-by-case basis via the use of bicistronic vectors in which cDNA of the transgene is followed by an IRES sequence and then by the cDNA for luciferase [15-17]. However, luciferase expression from bicistronic vectors is a reporter for the particular transgene expression rather than a marker of the ‘specific tissue content’ and, as such, should be used with caution for visualisation of the transgene-induced overgrowth of the malignant tissue. That especially holds true, if mice bearing ‘promoter-oncogene-IRES-luciferase’ transgene are intercrossed with another genetically modified strain. Also, markers for the ‘tissue content’ rather than the oncogenic transgene expression are more suitable, when the ‘oncogene’ acts in a paracrine manner and mammary tumours can be subsequently formed from the cells induced by the ‘oncogene’, but not expressing the ‘oncogene’ construct themselves. Moreover, vectors encoding ‘imageable’ molecules are unlikely to be introduced by direct genetic manipulation (e.g. microinjection) into already existing transgenic models, expressing only the oncogenic transgene under a tissue-specific promoter, such as MMTV-PyVT mice. Nevertheless, such models can still be rendered ‘imageable’, if interbred with another mouse strain already expressing an ‘imageable’ molecule under the same type of the promoter. Thus, the generation of such a mouse strain is vital for making many of the existing mammary tumour transgenic models applicable for advanced ***in vivo*** imaging techniques. In our project, we have generated a mouse strain expressing an enhanced firefly luciferase under a breast tissue-specific promoter and we present data documenting that it can be utilised as a new bioluminescent model for visualisation of mammary tumour development in transgenic mice.

## Materials and methods

### Generation of the MMTV-Luc2 transgenic construct

The pGL4.10[***luc2***] vector was purchased from Promega Corp. (Madison, WI). A MMTV-cFos-SV40 construct (Addgene plasmid 19259) was obtained from Dr. Philip Leder via Addgene Inc. (Cambridge, MA). The original cFos cDNA was replaced by Luc2 cDNA within the MMTV-SV40 construct using HindIII and XbaI (both enzymes from NEB, Ipswich, MA) digest, followed by T4 ligation (Promega). Prior to microinjection, the construct has been linearised by digestion with SpeI and SalI (NEB) enzymes, run on a 0.8% agarose gel with crystal violet, extracted from the gel via a Qiaex II gel extraction kit (Qiagen Inc., Valencia, CA), and then resuspended at a concentration of 1.2 ng/μl in EmbryoMax Injection Buffer (Millipore, Billerica, MA). In order to generate MMTV-Luc2 transgenic animals, pronuclear injections of the fertilised oocytes and embryo transfer to the pseudopregnant females was carried out within the UCD Conway Institute Biotechnical Services (CIBS) facility. Mice were genotyped using a PCR-based approach (see Additional file 1). Sequences of the primers used for genotyping of the MMTV-Luc2 mice are as follows: forward primer, 5’-ACAGAAACAACCAGCGCCATTCTG-3’; reverse primer, 5’-TCCAACTTGCCGGTCAGTCCTTTA-3’ (please see Additional file 1).

### Mice

Wild-type FVB/N mice and FVB/N-Tg(MMTV-PyVT)643Mul/J (MGI:2679595; further referred to as MMTV-PyVT mice) were purchased from Charles River UK (Margate, Kent, UK) and Jackson Laboratories (Bar Harbor, Maine), respectively. Mice were maintained and bred within the UCD CIBS and UCD Biomedical facilities under specific pathogen free (SPF) conditions with unrestricted access to food and drinking water. All protocols involving animals have been approved by UCD Animal Research Ethics Committee and carried out under the licence No. B100/4283 from the Irish Department of Health & Children. All transgenic work has been registered with the Environmental Protection Agency of Ireland (reg. No. G0310-01).

### Whole animal and *ex vivo* imaging

MMTV-Luc2 (heterozygous or homozygous) animals or double transgenic MMTV-Luc2 x MMTV-PyVT (both transgenes heterozygous, further referred to as MMTV-Luc2PyVT) animals were used for luciferase assay studies. All females subjected to imaging in this study were nulliparous. Prior to ***in vivo*** imaging, mice received intraperitoneal administration of luciferin in Dulbecco's Phosphate Buffered Saline (final dose of 150 mg/kg mouse body weight). Then, anesthetised animals (up to 5% isofluorane) were imaged using an IVIS Spectrum system (Caliper Life Sciences, Hopkinton, MA) to visualise luciferase-expressing tissues. In relation to imaging of non-oncogene-bearing animals, F/STOP parameter value 1 was used. In experiments with oncogene-bearing mice, F/STOP 2 was used for both oncogene-bearing and non-oncogene-bearing littermates. For ***ex vivo*** imaging, mice were injected with luciferin and imaged as stated above, subjected to euthanasia by cervical dislocation while under anaesthesia, and then internal organs or tumours were isolated and visualised by the IVIS Spectrum system. Imaging data were analysed using Living Image 3.2 software (Caliper). DLIT™ algorithm was used for 3D reconstitution.

### Statistical analysis

Prism 5 (GraphPad Software Inc, La Jolla, CA) software was used for statistical analysis, unless otherwise stated. Differences between the experimental groups in respect to total luminescent imaging during first measurement of whole body readout were analysed by a two-tailed unpaired Student’s ***t*** test (MS Excel 2007, Microsoft, Redmond, WA). Correlation between whole animal luminescent readout and tumour palpation was analysed using a Spearman correlation and linear regression curve fit tests. Difference in tumour-free survival between MMTV-***Luc2het*** and MMTV-Luc2PyVT females was analysed by Gehan-Breslow-Wilcoxon test. Results were considered to be statistically significant at p < 0.05.

## Results

### Identification of transgenic founders and generation of homozygous mice

**Figure 1:**
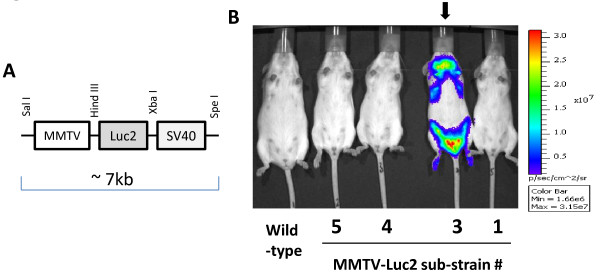
***Generation and bioluminescent imaging of MMTV-Luc2 mice. (A)*** Schematic of the transgenic construct used for generation of MMTV-Luc2 mice. ***(B)*** Whole-body luminescent imaging of representative nulliparous F1 females from sub-strains obtained by crosses of F0 female founders with wild-type FVB/N males. MMTV-Luc2 sub-strain #3 (***arrow***) was selected for further studies. ***Wild-type***: FVB/N female; ***1, 3, 4, 5***: numbers of the respective sub-strains (please refer to Additional file 1Figure S1). [Note: strain #2 was discontinued prior to imaging because of indication of embryonic lethality during transgene vertical transmission studies].

Following microinjections of the linearised transgene (Figure 1A) into fertilized oocytes and embryo transfer to pseudopregnant females, 35 pups were obtained. Ten of the pups were strongly positive for transgene presence in their genomic DNA, as assessed by PCR amplification (Additional file 1 Figure S1). Out of these, five females were chosen as potential founders (numbered #1-5) and crossed with wild-type FVB mice in order to assess germline transmission of the transgene. In case of founder #2, the percentage of transgene-positive offspring was markedly below the expected 50% Mendelian distribution, which suggested embryonic lethality, and thus the sub-strain #2 was discontinued. From the remaining four sub-strains, randomly chosen MMTV-Luc2-positive heterozygous females were subjected to whole animal luminescent imaging. As shown in Figure 1B, only one (#3) out of four imaged sub-strains presented clear mammary tissue-specific distribution of the luminescent signal. This sub-strain was selected for further interrogations. Another sub-strain (#5) showed low-level generalised skin luminescence, which was below the detection threshold under imaging conditions used in Figure 1B. Sub-strains #1 and #4 did not produce detectable luminescent signals via whole body luminescent imaging.

Additional file 1 Figure S2s demonstrates luminescent signal distribution within representative heterozygous mice from the MMTV-Luc2 #3 sub-strain (hereafter referred to as the ‘MMTV-Luc2’ strain). Using non-invasive whole animal imaging (Figure 1B and Additional file 1 Figure S 2), potent luminescence could be mainly seen in areas corresponding to the distribution of mammary tissue in females. Notably, regions corresponding to localisation of salivary glands were also strongly positive for luminescence, which is a classical distribution for MMTV-driven expression of the transgene [18,19]. After isolation of the internal organs (Figure 1D), luminescent signal of markedly lower magnitude was detected mainly from the spleen, lungs, thymus, and liver, as well as kidneys and intestine (Additional file 1 Figure S2). As expected, no gross alterations regarding breeding, mouse behaviour or anatomical parameters were observed in MMTV-Luc2 animals as compared to the wild-type littermates.

**Figure 2:**
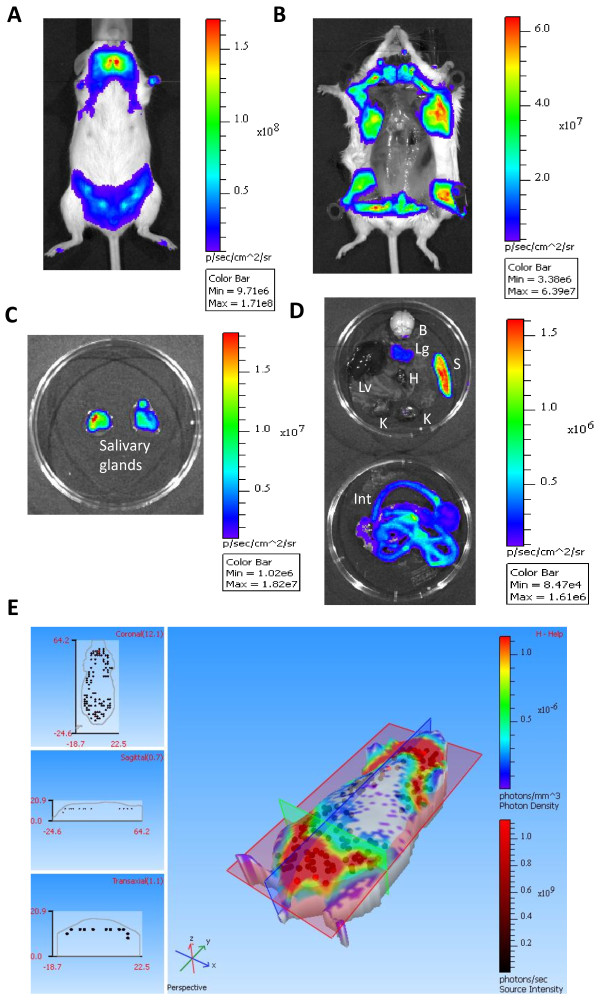
***Imaging of a representative nulliparous homozygous MMTV-Luc2 female mouse.*** [Note: A-D images were obtained from the same representative female; please refer to Additional file 1 Figures S3 and S 4 for additional examples] **(A)** Distribution of bioluminescent signal **via** whole body imaging. **(B)** Distribution of bioluminescent signal in whole-body autopsy. **(C)** Imaging of the isolated salivary glands. **(D)** Distribution of bioluminescent signal in isolated internal organs. ***B***: brain, ***H***: heart, ***In***t: intestine, ***K***: kidney, ***Lg***: lungs, ***Lv***: liver, ***S***: spleen. [Please note that the ranges of the luminescence readout between panels A and D are 100-fold different, as shown by the colour bar scales]. **(E)** Tomographic DLIT™ 3D reconstitution of the luminescent signal distribution [29] at the wavelength of 660 nm from a representative homozygous MMTV-Luc2 female (ventral view). Reconstituted sources of luminescent light are shown as spheres (please note a superficial localisation of the light sources). Left-hand panels represent slices acquired by slice planes shown on the right hand panel: coronal (red), sagittal (blue) and transaxial (green).

It must be stated, however, that heterozygous MMTV-Luc2 (MMTV-Luc2***het***) females presented noticeable variability in the strength and distribution of luminescent signal, as determined by non-invasive whole animal imaging (see below). Therefore, by intercrossing the MMTV-Luc2***het*** females and males, we have generated homozygous MMTV-Luc2 animals and used them for further breeding and maintenance of the strain. Again, no gross alterations were observed in homozygous MMTV-Luc2 animals. As shown in Additional file 1 Figure S3, homozygous females and males presented relatively uniform luciferase expression patterns as well as similar potency in respect to luminescence signal, as assessed by non-invasive whole animal imaging. More detailed representation of the luminescent signal distribution in the ***in vivo*** and ***ex vivo*** imaging of representative homozygous MMTV-Luc2 females is shown in Figure 2 and Additional file 1 Figure S4A-C,E,F. Figure 2E shows a 3-dimensional tomographic reconstitution of the signal distribution in a representative homozygous MMTV-Luc2 female. Whole body autopsy (Figure 2B) showed that the source of the signal from mammary gland regions was, as expected, subcutaneous, with the non-specific signal from the internal organs remaining below detection range under this exposure setting. Potent signal could be detected from isolated salivary glands (Figure 2C). After isolation of the internal organs (Figure 2D), luminescent signal of markedly lower magnitude was again detected mainly from the spleen, lungs, kidneys and intestine. Interestingly, the mouse intestine is capable of producing bioluminescent signal due to processes of digestion. In this study, we have specifically addressed the issue of intestinal luminescence by carrying out imaging of intestines isolated from a representative MMTV-Luc2 homozygous mouse without prior injection of luciferin. As shown in the revised Additional file 1 Figure S5, a low level luminescence was detected, which then increased roughly 10-fold after soaking the intestine with luciferin solution. That confirms the presence of spontaneous intestinal bioluminescence, but also suggests that the Luc2 transgene was also expressed in the intestine, most probably in the intestinal immune tissue, similar to the expression detected in the spleen. Nonetheless, as the background signal from internal organs, including intestine was negligible when compared to the potent signal from salivary and mammary glands (Figures 2B-D), which was further dramatically amplified in developing tumours (see below), we do not find maintenance of MMTV-Luc2 mice on a specialised diet necessary.

Also, according to the blinded pathological assessment, there was no evidence of histological alterations in mammary gland structure in the homozygous MMTV-Luc2 versus age-matched control mice, with both of them showing normal mammary gland growth and development (Additional file 1 Figure S4D).

### Monitoring of the mammary tumour development

**Figure 3:**
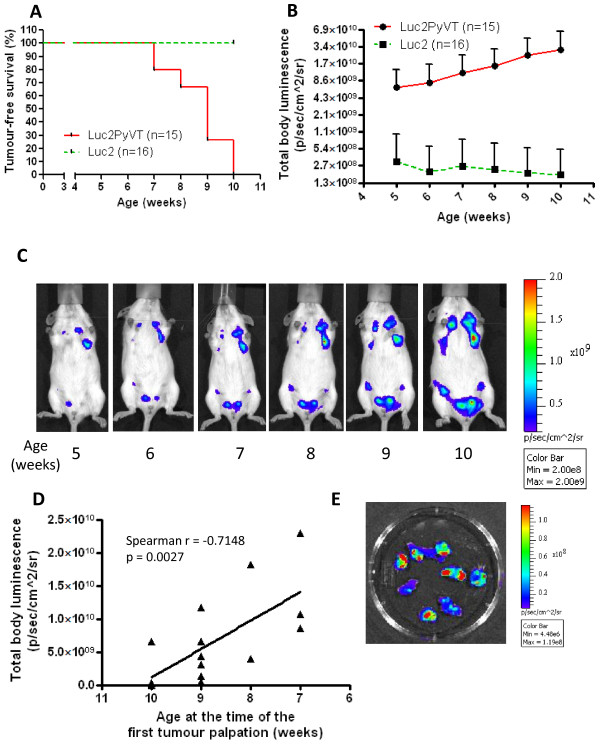
***Evaluation of development and progression of spontaneous mammary tumours in MMTV-Luc2PyVT mice*versus*MMTV-Luc2het controls. (A)*** Evaluation of tumour development by palpation in double heterozygous MMTV-Luc2PyVT female mice **versus** MMTV-Luc2***het*** females. ***(B)*** Evaluation of the total body luminescent signal in MMTV-Luc2PyVT female mice **versus** MMTV-Luc2***het*** females between 5-10 weeks of age. ***Points***: mean values + standard deviations ***(C)*** Longitudinal observation of the localisation and intensity of luminescent signal in a representative MMTV-Luc2PyVT female between 5-10 weeks of age. ***(D)*** Correlation between the intensity of total body luminescence readout at 5 weeks of age and the age of development of the first palpable tumour in MMTV-Luc2PyVT female mice. ***Line***: linear regression curve fit (r^2^ = 0.4759; p = 0.0044) ***(E) Ex vivo*** luminescent imaging of mammary tumours isolated from the representative MMTV-Luc2PyVT female [shown in panel ***(C)***] at the age of 10 weeks.

Having established the MMTV-Luc2 strain, we assessed its utility as a tool for monitoring of mammary tumour development and progression in oncogene-bearing transgenic mice. To this end, homozygous MMTV-Luc2 females were interbred with MMTV-PyVT heterozygous males. Among 31 littermates from these crosses, 16 pups were identified by PCR as MMTV-Luc2***het***, with the remaining 15 identified as MMTV-Luc2PyVT double transgenic (and double heterozygous) mice. Then, mice were subjected to non-invasive whole body luminescent imaging between the ages of 5 up to 10 weeks. The age of each animal at the time of each measurement was approximated to full weeks (median age at the time of the first measurement was 35 days, range 34-38 days). Simultaneously, mammary tumour development was monitored in mice by observation and palpation. As shown in Figure 3A, all PyVT-bearing mice developed palpable tumours by the age of 10 weeks, which corresponds to the described phenotype of the MMTV-PyVT transgenic strain [8,19]. No tumours were detected in MMTV-Luc2***het*** animals throughout the experiment. During the initial luminescent imaging at 5 weeks of age, the MMTV-Luc2***het*** control animals presented with variable distribution and potency of luminescent signal (Additional file 1 Figure S6A). Conversely, virtually all MMTV-Luc2PyVT littermates have shown foci of potent luminescent signal, which correlated with the localisation of mammary tissue and indicated regional pre-malignant or early malignant overgrowth of mammary epithelium in these spots (Additional file 1 Figure S6B and C), while, notably, none of the animals within the experiment produced a palpable tumour by the age of 5 weeks. On average, total body luminescence from the MMTV-Luc2PyVT animals during the first measurement exceeded that from control MMTV-Luc2***het*** mice by 21.01-fold (Figure 3B, p = 0.003 at 5-week time point). The mean total body luminescent readout from MMTV-Luc2PyVT mice increased exponentially in subsequent weeks (Figure 3B and 3C; please note the log_2_ scale of the Y axis in Figure 3B), whereas the readout from MMTV-Luc2***het*** controls did not show tendency to increase with age. Importantly, there was a significant inverse correlation between the potency of the first luminescent total body readout and the age of the first palpable tumour development in MMTV-Luc2PyVT mice, i.e. mice with higher luminescent signal at the age of 5 weeks tended to develop palpable tumours sooner in the following weeks (Figure 3D). To evaluate the ability of individual tumours to produce luminescent light, three MMTV-Luc2PyVT mice were randomly selected at the end of the experiment for ***post mortem*** isolation of tumours for ***ex vivo*** imaging. As shown in Figure 3E and Additional file 1 Figure S7A, all isolated tumours produced detectable luminescent signal, although variable in its potency. This observation corresponds to the previously reported phenomenon of variable expression of MMTV promoter-driven transgenes across mammary epithelium [19]. In histological evaluation, the tumours were classified, as expected, as adenocarcinomas, malignant tumours of glandular epithelium (Additional file 1 Figure S7B). This corresponds with pathological observations from the initial publication regarding the MMTV-PyVT mouse model [8].

## Discussion

In this work, we report successful generation of a new transgenic mouse strain expressing an enhanced form of firefly luciferase (Luc2) in a mammary tissue-specific manner. We also report successful application of this new strain for visualisation of primary tumour development and longitudinal monitoring of local tumour progression in oncogene-bearing transgenic animals.

Improvement of currently existing transgenic animal models for breast cancer is a pressing issue and has been addressed by different approaches in the past. Conceptually, MMTV-Luc2 mouse model stands in line with previously generated models of luciferase expression driven by other tissue-specific promoters, which are currently available from Taconic Farms, Inc. (Hudson, NY). These models include such strains as FVB/N-Tg(Vegfr2-luc)-Xen (vascular-specific expression [20]) or FVB/N-EL1-Luc/EL1-Tag (pancreatic expression).

Theoretically, another strain available from Taconic, the LucRep (FVB) mice [21], can be engineered for mammary tissue-specific expression using a Cre-activated system [22]. It has been previously shown that Cre-activated luminescence permits, for instance, a temporally and spatially controlled expression of responder genes in embryonic and multiple adult tissues [23]. Therefore, from a mechanistic perspective, using a Cre-activated system would provide decreased variability of the model system as compared to MMTV-driven expression. It must be considered, however, that Cre-activated luminescence brings the need for using yet another mouse strain (e.g. WAP-Cre [22]) within the crossbreeding schedule. Conversely, one of the strengths of the MMTV-Luc2 mice lies in the increased feasibility of this system over Cre-activated models; indeed, the MMTV-Luc2 model could be found desirable for some transgenic projects, especially ones utilising non-Cre-activated oncogenic strains, such as MMTV-PyVT. It is also important to mention that using the Cre-activated system can also lead to mosaic expression profile in some instances [19]. In summary, while certain advantages of Cre-activated systems are obvious, MMTV-Luc2 mice can provide a practical alternative to these models. ODD-luciferase mice [24,25] are another universal strain with potential use for imaging of mammary tumours in transgenic animals. In this model, expression of luciferase is induced by hypoxic conditions within tumours. However, ***in vivo*** imaging in ODD-luciferase strain has shown significant generalised background luminescence of the body [25], as hypoxia is intrinsic in some tissues under normal conditions, which can hamper exact localising of early overgrowth of mammary tissue **via*in vivo*** imaging. Notably, this task can be successfully achieved by the use of MMTV-Luc2 model (Figure 3C and Additional file 1 Figure S6B)

Our model shows also close conceptual similarities with previously generated MMTV- or WAP- GFP mice, which are mouse strains expressing green fluorescent protein (GFP) under various mammary tissue-specific promoters [19]. GFP-based imaging has been also successfully used for longitudinal studies of mammogenesis [26]. However, although successfully generated, the applicability of GFP-based systems is of very limited value for whole animal ***in vivo*** imaging, mainly because of autofluorescence-related problems and low tissue penetration of the light emitted by GFP. Conversely, detection of bioluminescence from cells expressing firefly luciferase is considered the most sensitive technique for optical imaging ***in vivo***[11], as light produced after the reaction catalysed by luciferase penetrates the tissues with high efficacy. The issue of autofluorescence is also alleviated, as bioluminescence does not require the excitation light. Hence, our model holds substantial advantages over GFP-based systems.

It is important to point out that the MMTV-Luc2 model by design is not expected to be capable of detecting metastases (e.g. into the lungs) directly in living transgenic animals, primarily because of the robust localisation of mammary tissue in female mice. In result, the background signal from superficially located mammary tumours and also healthy mammary tissue would set up the imaging threshold too high for potential detection of metastases in whole body imaging ***in vivo***. Therefore, we wish to recommend the usage of the MMTV-Luc2 mice mainly for monitoring the growth of primary tumours, at least with the currently available bioluminescent imaging systems.

Another issue related to the applicability of the MMTV-Luc2 model system is the evidence from previous studies that the MMTV-driven expression of the transgene is primarily active in luminal epithelial cells rather than basal cells in the gland (reviewed in [27]). Therefore, while MMTV-Luc2 mice appear relevant for imaging of other MMTV-driven oncogenic model systems, as exemplified by the MMTVPyVT model in this work, the applicability of MMTV-Luc2 imaging in basal-type breast cancer models (such as BRCA1-knockout mice [28]) is yet to be studied.

As mentioned above, the MMTV-Luc2 model has been primarily designed for rendering the currently available MMTV-oncogene-based strains applicable for advanced luminescent imaging. Such enhancement greatly increases the capability of monitoring local tumour development and progression, but potentially also response to therapies in such models. We present an example of such an application by intercrossing the MMTV-Luc2 mice with the MMTV-PyVT strain. However, what must be mentioned is that luciferase-based quantification of the signal is not primarily designed to not be used for direct quantification of the tumour burden in living animals, but rather as an indicative marker. One of the reasons for this fact is the well established phenomenon of mosaic expression of the MMTV-driven transgene, especially in heterozygous animals. Indeed, in the MMTV-Luc2 model, we do see some differences in production of the luminescent signal among the tumours taken out from the MMTV-Luc2PyVT females (Figure 3E and Additional file 1 Figure S7A). Secondly, MMTV promoter activation is known to be modulated by steroid and peptide hormones that, in turn, can alter a linear correlation between cell number and the potency of luciferase signal. Therefore, we would like to recommend the MMTV-Luc2 imaging as supportive tool to manual methods of tumour monitoring. At the same time, we would like to strongly point out that homozygous MMTV-Luc2 females express a much more unified pattern of luminescent signal considering the promoter being used, which indicates they can be feasibly applicable for tumour visualisation purposes in transgenic models for breast cancer.

For future applications, as the MMTV-Luc2 homozygous mice are viable and fertile, this strain can be also used instead of wild-type FVB mice as a primary strain for generating new models incorporating mammary tissue-specific expression of a molecule of choice. Ideally, bicistronic vectors encoding the molecule of interest (most frequently a putative oncogene) and fluorescent markers (to highlight oncogenic transgene expression) would be introduced into MMTV-Luc2 mice and then visualised in a dual-modality manner for co-localisation of fluorescence and luminescence. This would provide information on both levels of transgene expression (by fluorescence) and potential local changes in mammary tissue ‘content’ (by luminescence) in these new models. Importantly, the use of the homozygous MMTV-Luc2 mice in such systems will decrease the intra-strain variability of tissue-specific Luc2 expression in comparison to imaging of heterozygous MMTV-Luc2 mice. This, in turn, would correspond to the current ethical considerations regarding studies involving animals, i.e. our model will facilitate reduction in the number of transgenic animals per group necessary for obtaining statistically significant results.

## Conclusions

In summary, our project has provided a novel transgenic strain for early detection and monitoring of mammary tumour development in transgenic mice. Generation of this mouse strain is vital for making many of the existing mammary tumour transgenic models applicable for advanced ***in vivo*** imaging techniques. It can also serve as a technology platform and a background strain for further development of new models.

## Abbreviations

BLG, Beta-lactoglobulin; CT, Computed tomography; GFP, Green fluorescent protein; IRES, Internal ribosomal entry site; MMTV-LTR, Mammary tumour virus long terminal repeat; Luc, Luciferase; ODD, Oxygen-dependent degradation domain; PET, Positron emission tomography; PyVT, Polyomavirus middle T antigen; SPECT, Single photon emission computed tomography; WAP, Whey acidic protein.

## Misc

William M Gallagher and Radoslaw Zagozdzon share equal senior authorship

## Competing interests

The authors declare that they have no competing interests.

## Authors’ contributions

AMZ performed genotyping, ***in vivo*** and ***ex vivo*** imaging, ***post mortem*** preparation of tissues, participated in histology procedures and analysis of the imaging results as well as contributed to preparation of the draft of the manuscript; POL carried out statistical analyses, histology procedures and contributed to preparation of the draft of the manuscript; JJC carried out histology procedures and analysed the histological material; JC participated in design of the study and provided insights into translational perspective of this project; WMG participated in design and coordination of the study and contributed to the final version of the manuscript; RZ conceived the idea, generated the transgenic construct, participated in design and coordination of the study, ***in vivo*** and ***ex vivo*** imaging, analysed the imaging results and contributed to the final version of the manuscript. All authors read and approved the final version of the manuscript.

## Pre-publication history

The pre-publication history for this paper can be accessed here:

http://www.biomedcentral.com/1471-2407/12/209/prepub

## Supplementary Material

Additional file 1 **Figure S1. **Identification of potential founders from F0 pups born after microinjections of MMTV-Luc2 construct. Ten mice (blue arrows) were identified by PCR as positive for transgene presence, and five females (green arrows) were selected as potential founders of the MMTV-Luc2 sub-strains #1-5. DNA marker used: 2-Log DNA Ladder (0.1–10.0 kb) (NEB). **Figure S2.** Whole-body luminescent imaging (upper panels) and *ex vivo* imaging of isolated internal organs (lower panels) of three representative MMTV-Luc2*het* virgin females. *B*: brain, *H*: heart, *Int*: intestine, *K*: kidney, *Lg*: lungs, *Lv*: liver, *S*: spleen, *Th*: thymus. **Figure S3.** Distribution of luminescent signal from whole-body imaging of homozygous MMTV-Luc2 littermates. (A) virgin females; (B) males. Mice were of 101 days of age at the time of imaging. **Figure S4.** Presentation of a representative homozygous MMTV-Luc2 virgin female (A) Distribution of luminescent signal in a whole-body imaging; (B) ex-vivo whole body necropsy; (C) *ex vivo* imaging of isolated internal organs; B: brain, H: heart, Int: intestine, K: kidney, Lg: lungs, Lv: liver, S: spleen, Th: thymus; (D) representative histological image of the mammary gland of MMTV-Luc2 virgin female. Haematoxylin and eosin stained section of mammary tissue revealing randomly dispersed solitary variable-sized ductular structures embedded in adipose tissue (Bar= 100 μm). (E) and (F) Tomographic surface reconstitution of the luminescent signal from a representative homozygous MMTV-Luc2 female from ventral and dorsal views, respectively. **Figure S5.** Imaging of the intestine from a representative homozygous MMTV-Luc2 female mouse. The intestine was isolated without prior injection of the luciferin solution to the mouse, 3 therefore the left panel represents spontaneous luminescent signal from the intestine. Then, luciferin solution (300 μg/ml) was applied in drops on the isolated organ and imaging was repeated (right panel). **Figure S6.** Localisation of the luminescent signal in the whole-body luminescent imaging of F1 females from crosses between homozygous MMTV-Luc2 females and MMTV-PyVT*het* males. (A) MMTV-Luc2*het* females (B) Double transgenic and double heterozygous MMTV-Luc2PyVT females. [Note: in panels (A) and (B) mice were imaged for one second and the automatic imaging colour scale adjustments provided by the IVIS Spectrum system were used. The colour bars were removed for the sake of clarity of the figure.] (C) Image obtained with mouse number 27 after setting the exposure time to 20 sec. **Figure S7.** (A) Whole-body (upper panels) and e*x vivo* luminescent imaging of mammary tumours (lower panels) isolated from two representative MMTV-Luc2PyVT females at the age of 10 weeks. (B) Histopathological assessment showing evidence of mammary adenocarcinoma formation in a representative MMTV-Luc2PyVT female at the age of 10 weeks. Haematoxylin and eosin stained sections of mammary tumour revealing a multinodular densely cellular mass with cells arranged in sheets and acinar patterns. Increased magnification reveals moderate cell pleomorphism, prominent mitosis and acinar structure formations. (Left-hand image – Bar=500 μm; right-hand image – Bar=25 μm). Click here for file
