# Supplementary material for: Generation of a new bioluminescent model for visualisation of mammary tumour development in transgenic mice
Source: BMC Cancer. 2012 May 30;12:209. doi: 10.1186/1471-2407-12-209 (PMC3411433; doi:10.1186/1471-2407-12-209)

Supplementary Materials and Methods

Genotyping. For DNA extraction, mouse ear snips were incubated for approximately 16 hr in PBND buffer (50 mM KCl, 10 mM Tris HCl pH 8.3, 2.5 mM $MgCl_2$, 0.1 mg/ml gelatin, 0.45% v/v NP40, 0.45% v/v Tween-20) containing proteinase K (50 µg/ml, Promega). Then, proteinase K was inactivated by incubation for 5 min at 95ºC. Subsequently, samples were centrifuged at 20,000 *g* for 1 min and the supernatant was collected. PCR amplification was used for detection of the transgene in somatic DNA. One µl of each tissue sample supernatant was used per PCR reaction. GoTaq DNA polymerase was purchased from Promega and used according to the manufacturer's instructions. PCR conditions were as follows: 95ºC for 1 min, 30x [95ºC for 30 s, 60ºC for 30 s, 72ºC for 40 s], 72ºC for 2 min. MMTV-PyVT mice were genotyped with the use of GoTaq Hot Start polymerase (Promega) according to the instructions provided by Jackson Laboratories. PCR products were run on 1.2% agarose gels with ethidium bromide, visualised using a GENEGenius gel imaging system, and then analysed with Genesnap software (both from Syngene, Cambridge, England).

Histopathology. After *ex* vivo imaging, internal organs and tumours were fixed in 10% neutral buffered formalin (Sigma-Aldrich) for 24-hr. Tissue was then processed using a Leica TP1020 tissue processor (Leica Microsystems, Wetzlar, Germany) and blocked in paraffin, sectioned and stained with haematoxylin and eosin.

Supplementary Figure Legends

Supplementary Figure 1. **Identification of potential founders from F0 pups born after microinjections of MMTV-Luc2 construct.** Ten mice (blue arrows) were identified by PCR as positive for transgene presence, and five females (green arrows) were selected as potential founders of the MMTV-Luc2 sub-strains #1-5. DNA marker used: 2-Log DNA Ladder (0.1–10.0 kb) (NEB).

Supplementary Figure 2. **Whole-body luminescent imaging (upper panels) and *ex vivo* imaging of isolated internal organs (lower panels) of three representative MMTV-Luc2*het* virgin females.** *B*: brain, *H*: heart, *Int*: intestine, *K*: kidney, *Lg*: lungs, *Lv*: liver, *S*: spleen, *Th*: thymus.

Supplementary Figure 3. **Distribution of luminescent signal from whole-body imaging of homozygous MMTV-Luc2 littermates. (A)** virgin females; **(B)** males. Mice were of 101 days of age at the time of imaging.

Supplementary Figure 4. **Presentation of a representative homozygous MMTV-Luc2 virgin female (A)** Distribution of luminescent signal in a whole-body imaging**; (B)** ex-vivo whole body necropsy; **(C)** *ex vivo* imaging of isolated internal organs; B: brain, H: heart, Int: intestine, K: kidney, Lg: lungs, Lv: liver, S: spleen, Th: thymus; **(D)** representative histological image of the mammary gland of MMTV-Luc2 virgin female. Haematoxylin and eosin stained section of mammary tissue revealing randomly dispersed solitary variable-sized ductular structures embedded in adipose tissue (Bar= 100 μm). **(E)** and **(F)** Tomographic surface reconstitution of the luminescent signal from a representative homozygous MMTV-Luc2 female from ventral and dorsal views, respectively.

Supplementary Figure 5. **Imaging of the intestine from a representative homozygous MMTV-Luc2 female mouse.** The intestine was isolated without prior injection of the luciferin solution to the mouse,

therefore the left panel represents spontaneous luminescent signal from the intestine. Then, luciferin solution (300 µg/ml) was applied in drops on the isolated organ and imaging was repeated (right panel).

Supplementary Figure 6. **Localisation of the luminescent signal in the whole-body luminescent imaging of F1 females from crosses between homozygous MMTV-Luc2 females and MMTV-PyVT*het* males. (A)** MMTV-Luc2*het* females **(B)** Double transgenic and double heterozygous MMTV-Luc2PyVT females. [Note: in panels **(A)** and **(B)** mice were imaged for one second and the automatic imaging colour scale adjustments provided by the IVIS Spectrum system were used. The colour bars were removed for the sake of clarity of the figure.] **(C)** Image obtained with mouse number 27 after setting the exposure time to 20 sec.

Supplementary Figure 7. **(A)** Whole-body (upper panels) and e*x vivo* luminescent imaging of mammary tumours (lower panels) isolated from two representative MMTV-Luc2PyVT females at the age of 10 weeks. **(B)** Histopathological assessment showing evidence of mammary adenocarcinoma formation in a representative MMTV-Luc2PyVT female at the age of 10 weeks. Haematoxylin and eosin stained sections of mammary tumour revealing a multinodular densely cellular mass with cells arranged in sheets and acinar patterns. Increased magnification reveals moderate cell pleomorphism, prominent mitosis and acinar structure formations. (Left-hand image – Bar=500 µm; right-hand image – Bar=25 µm)

Supplementary Figure 1

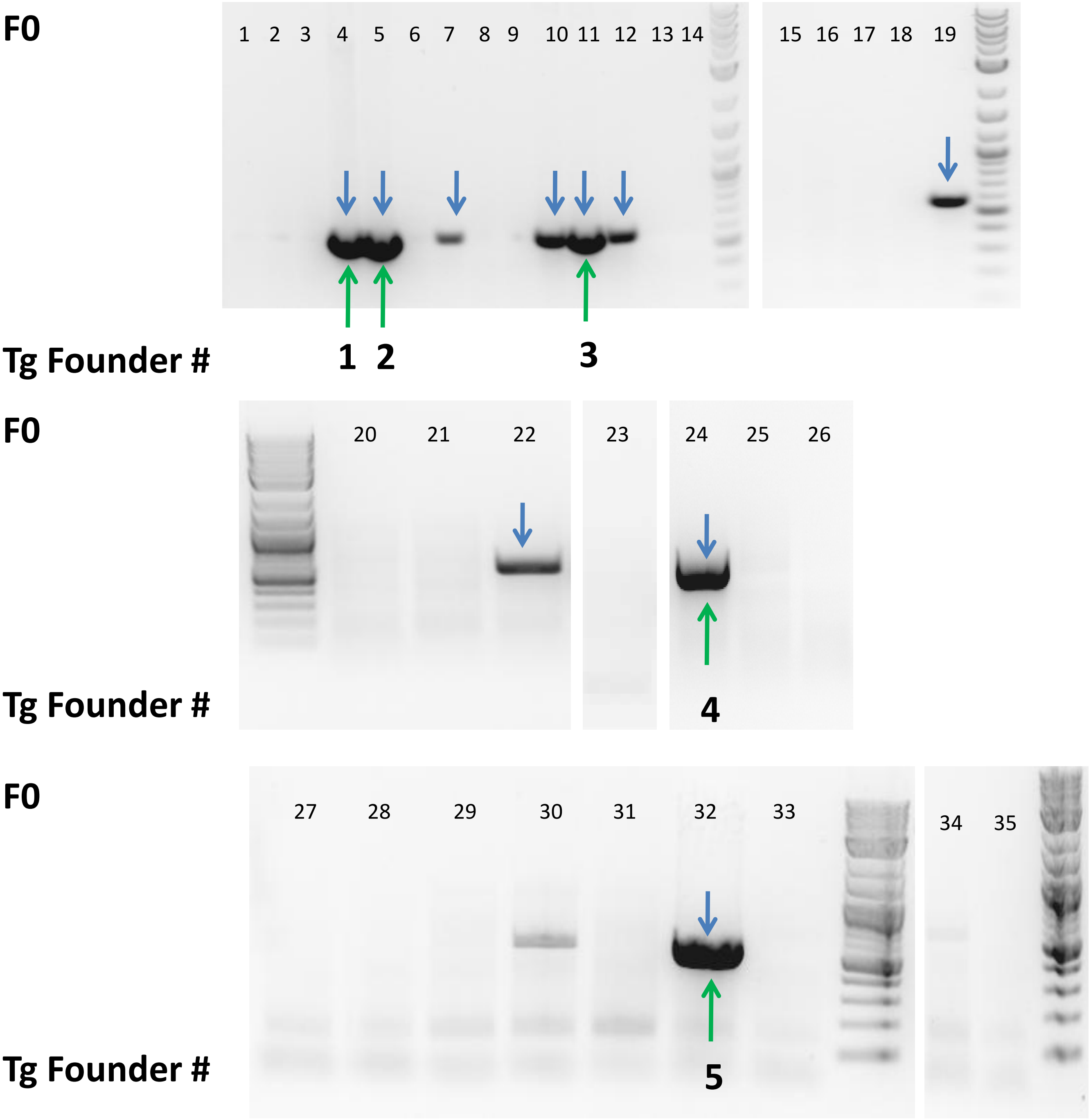

Supplementary Figure 2

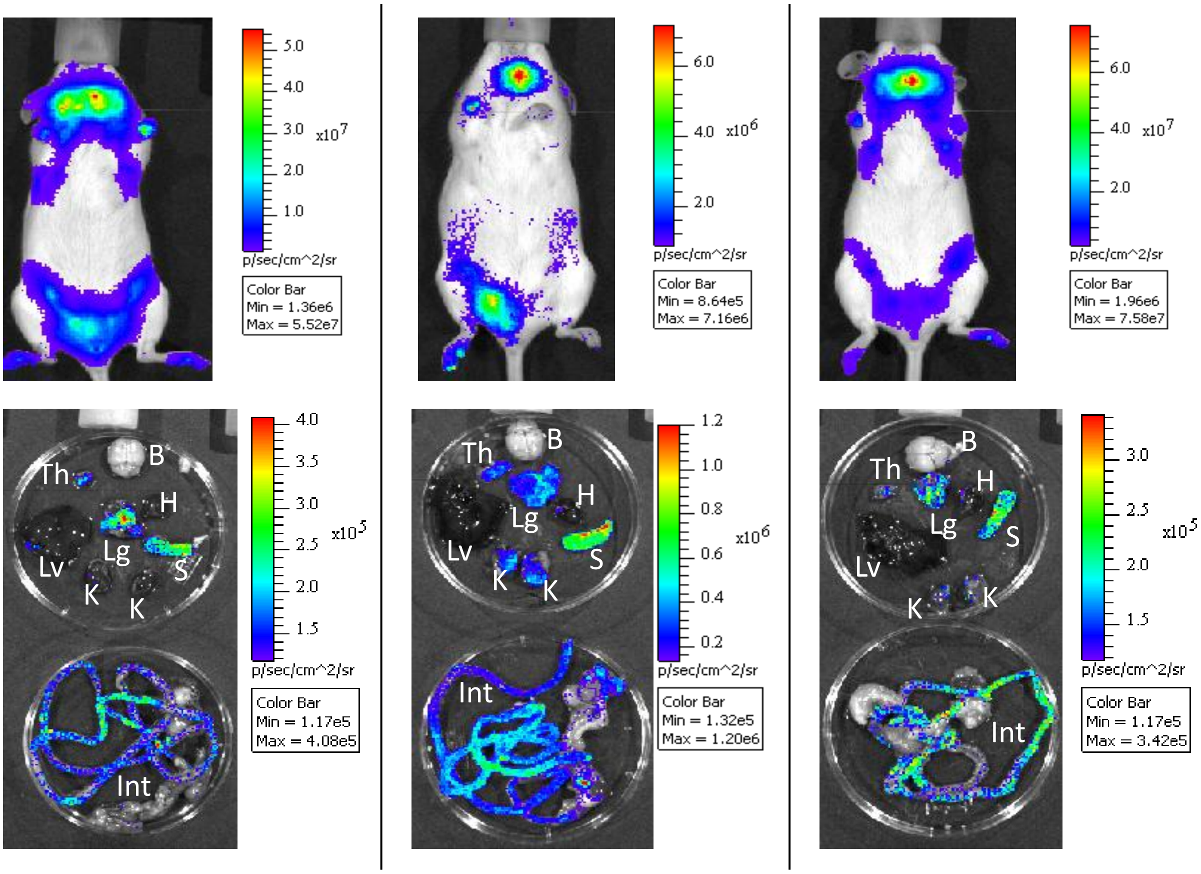

Supplementary Figure 3

A

Homozygous MMTV-Luc2 females

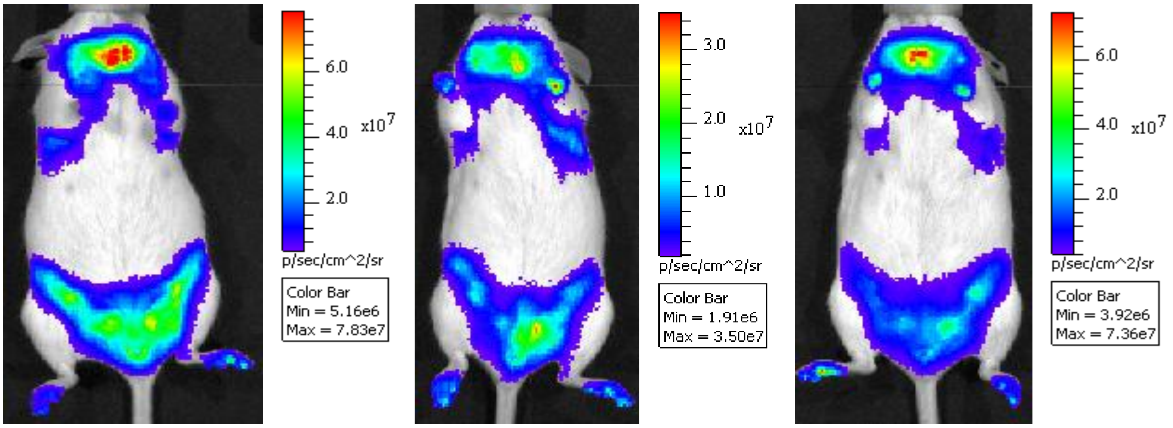

B

Homozygous MMTV-Luc2 males

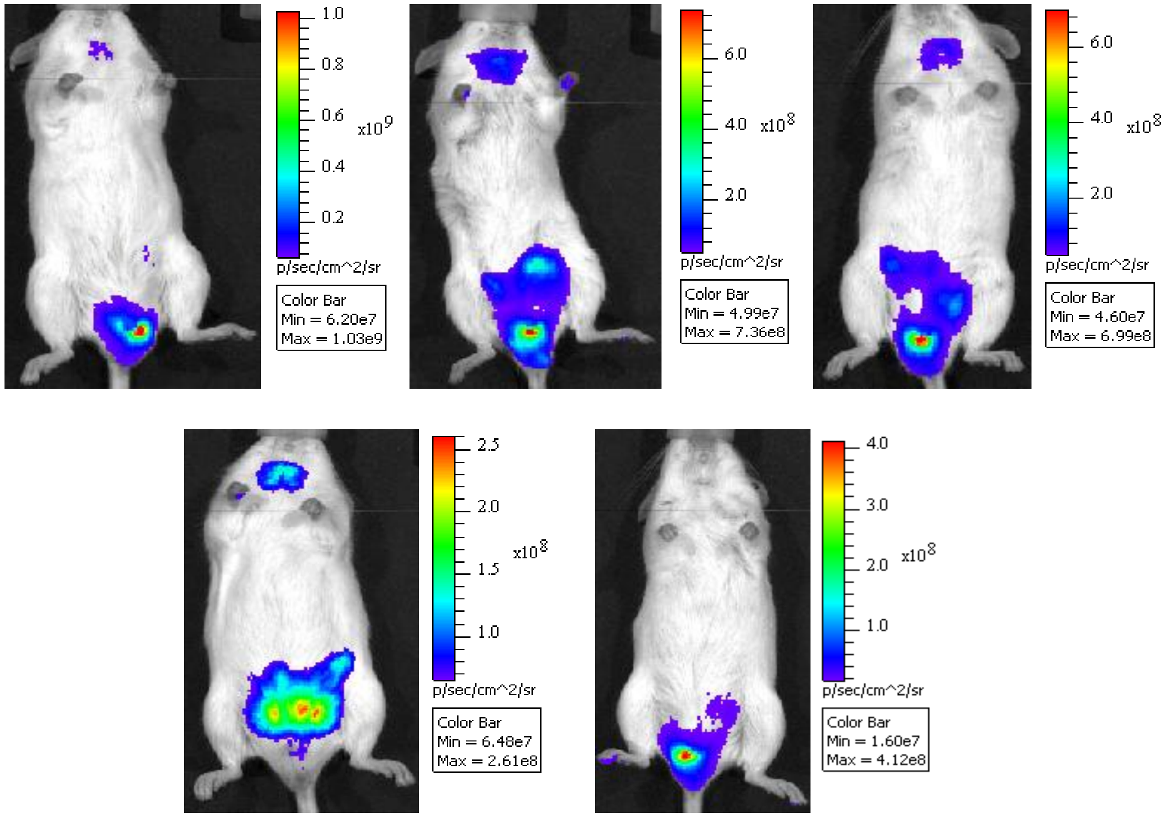

Supplementary Figure 4

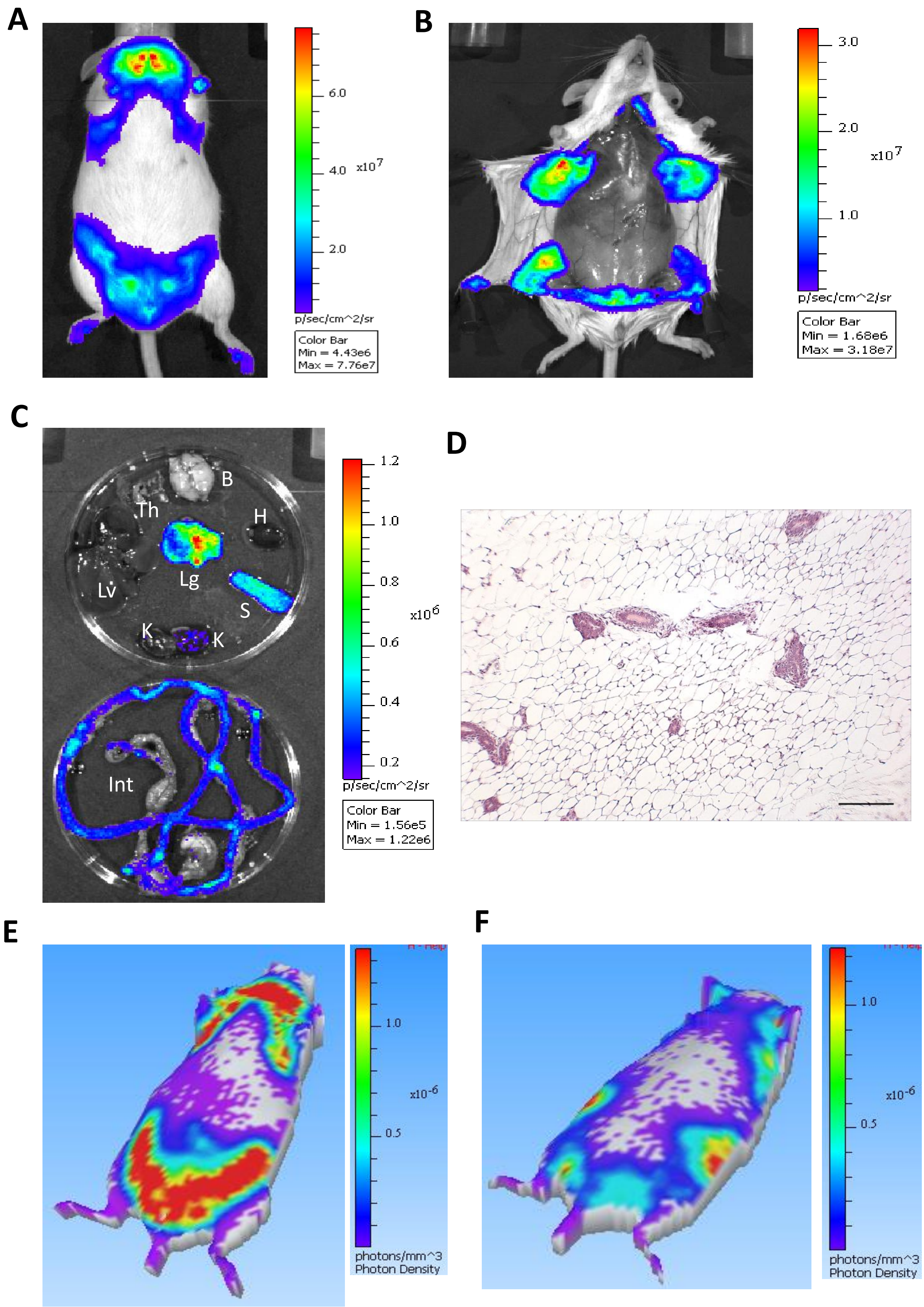

Supplementary Figure 5

Intestine

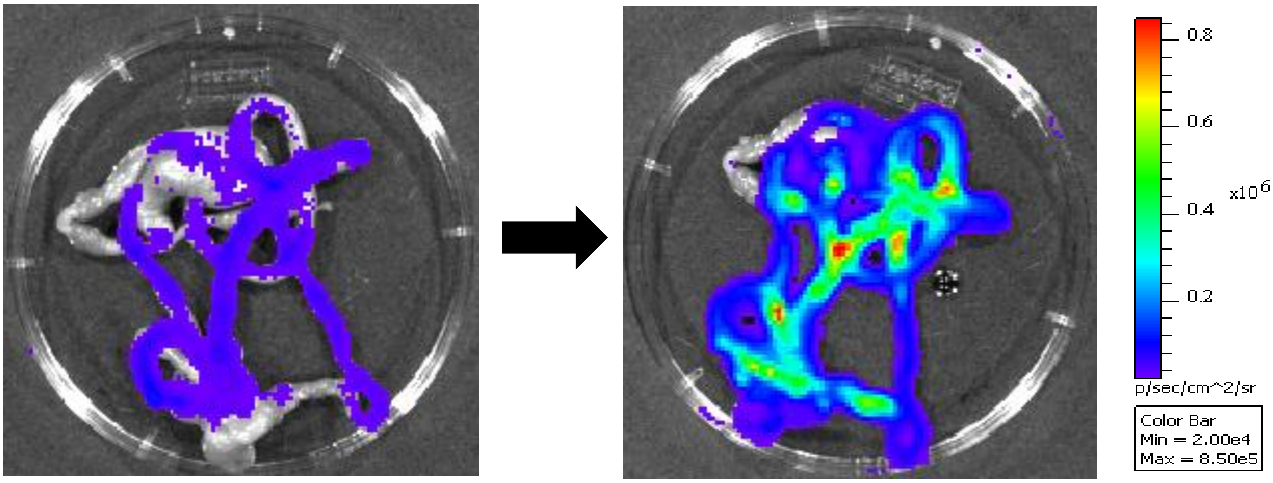

No luciferin

+ luciferin

Supplementary Figure 6

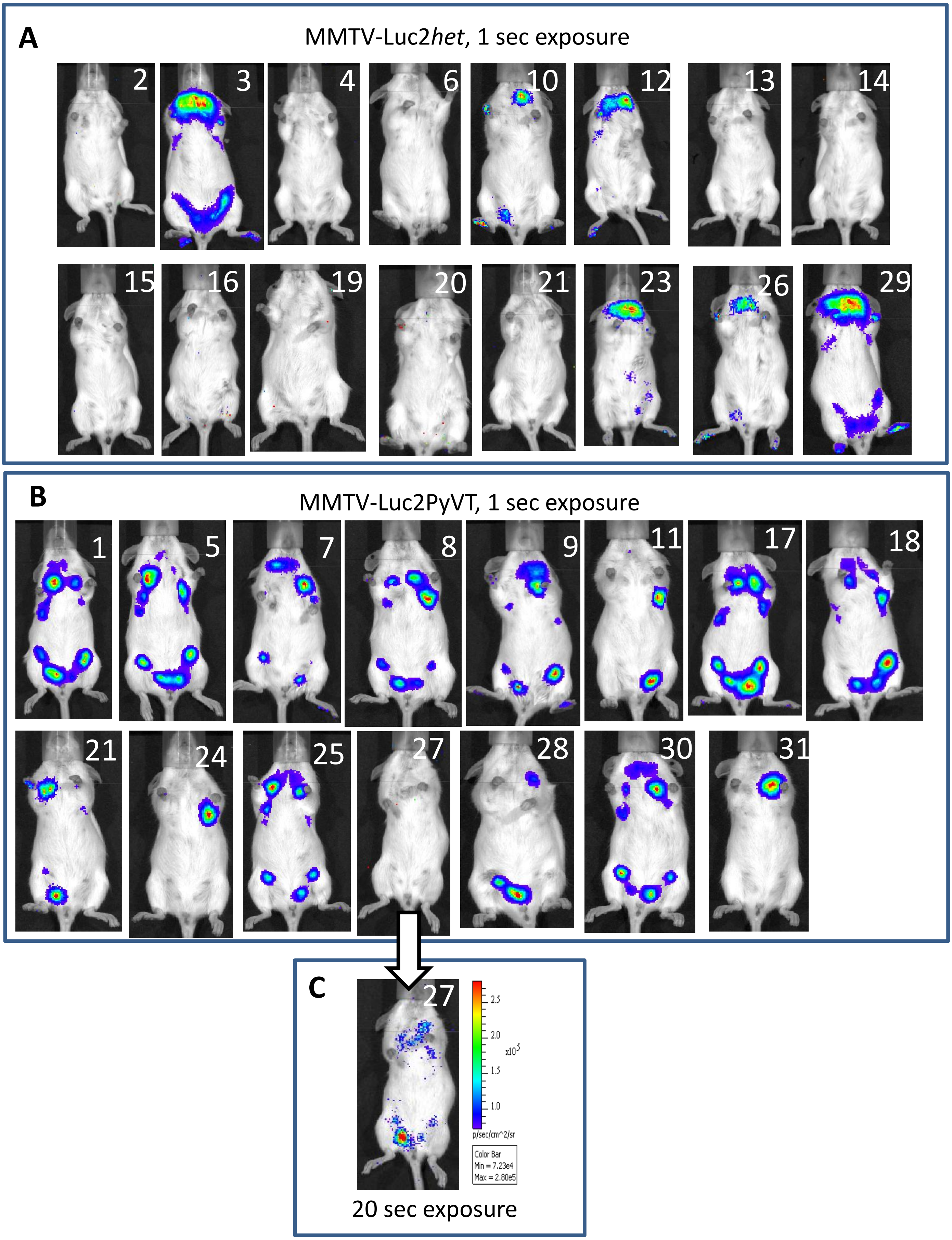

Supplementary Figure 7

A

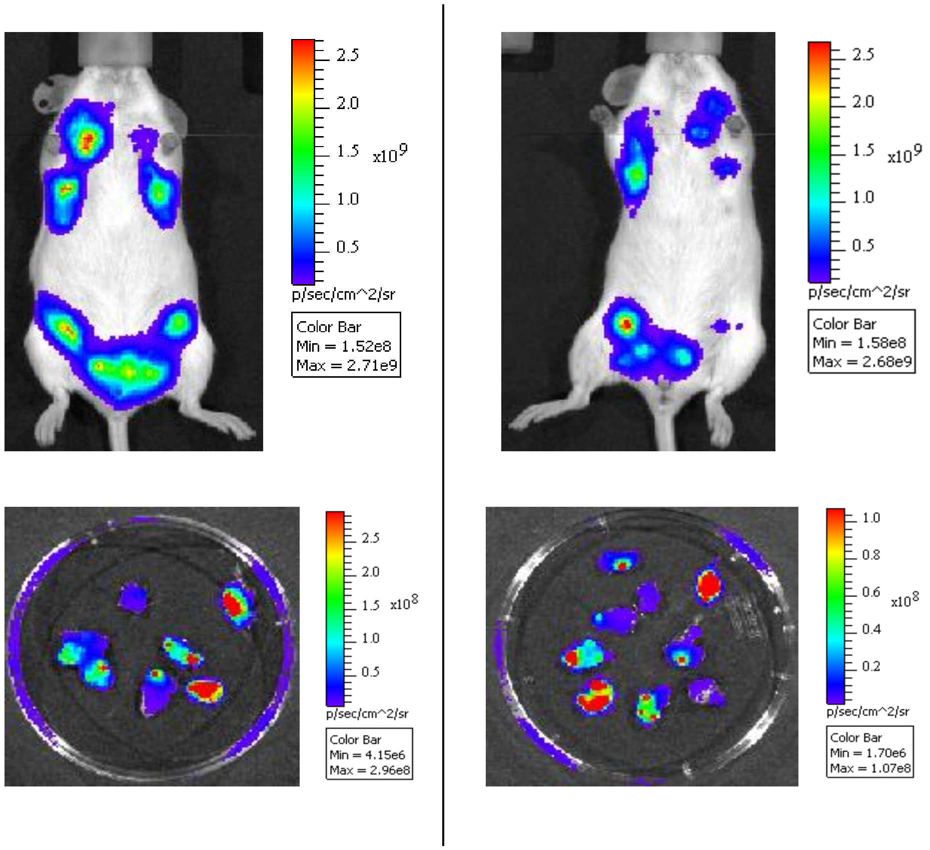

B

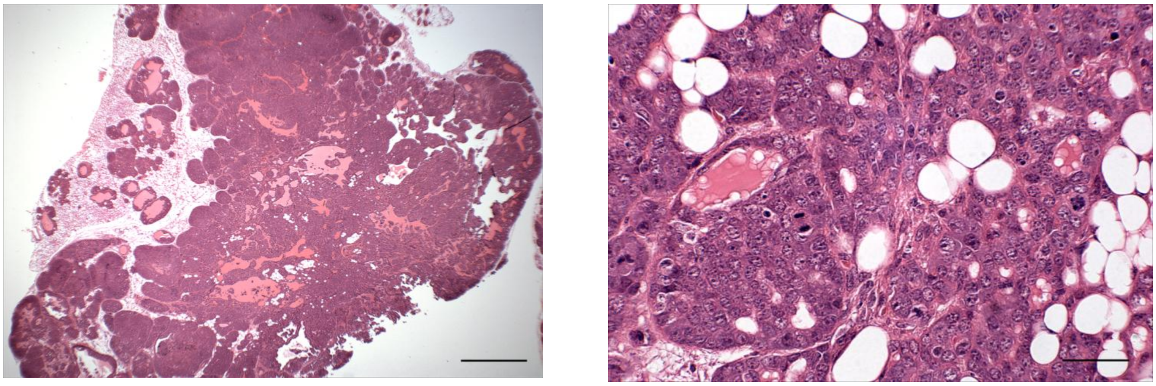

Supplement: Additional file 1 — Figure S1. Identification of potential founders from F0 pups born after microinjections of MMTV-Luc2 construct. Ten mice (blue arrows) were identified by PCR as positive for transgene presence, and five females (green arrows) were selected as potential founders of the MMTV-Luc2 sub-strains #1-5. DNA marker used: 2-Log DNA Ladder (0.1–10.0 kb) (NEB). Figure S2. Whole-body luminescent imaging (upper panels) and ex vivo imaging of isolated internal organs (lower panels) of three representative MMTV-Luc2het virgin females. B: brain, H: heart, Int: intestine, K: kidney, Lg: lungs, Lv: liver, S: spleen, Th: thymus. Figure S3. Distribution of luminescent signal from whole-body imaging of homozygous MMTV-Luc2 littermates. (A) virgin females; (B) males. Mice were of 101 days of age at the time of imaging. Figure S4. Presentation of a representative homozygous MMTV-Luc2 virgin female (A) Distribution of luminescent signal in a whole-body imaging; (B) ex-vivo whole body necropsy; (C) ex vivo imaging of isolated internal organs; B: brain, H: heart, Int: intestine, K: kidney, Lg: lungs, Lv: liver, S: spleen, Th: thymus; (D) representative histological image of the mammary gland of MMTV-Luc2 virgin female. Haematoxylin and eosin stained section of mammary tissue revealing randomly dispersed solitary variable-sized ductular structures embedded in adipose tissue (Bar= 100 μm). (E) and (F) Tomographic surface reconstitution of the luminescent signal from a representative homozygous MMTV-Luc2 female from ventral and dorsal views, respectively. Figure S5. Imaging of the intestine from a representative homozygous MMTV-Luc2 female mouse. The intestine was isolated without prior injection of the luciferin solution to the mouse, 3 therefore the left panel represents spontaneous luminescent signal from the intestine. Then, luciferin solution (300 μg/ml) was applied in drops on the isolated organ and imaging was repeated (right panel). Figure S6. Localisation of the luminescent signal in th [file 1471-2407-12-209-S1.pdf]
